# Effects of Sprifermin, IGF1, IGF2, BMP7, or CNP on Bovine Chondrocytes in Monolayer and 3D Culture

**DOI:** 10.1002/jor.24491

**Published:** 2019-10-29

**Authors:** Sylvia Müller, Sven Lindemann, Anne Gigout

**Affiliations:** ^1^ Osteoarthritis Research, Merck KGaA Frankfurter Strasse 250 Darmstadt 64293 Germany

**Keywords:** growth factor, DMOAD, cartilage, sprifermin, osteoarthritis

## Abstract

One possible approach to treat osteoarthritis (OA) is to counteract cartilage degeneration with anabolic compounds that stimulate chondrocyte proliferation and/or extracellular matrix (ECM) production. Several molecules including sprifermin (recombinant human fibroblast growth factor [FGF18]), insulin‐like growth factor‐1 [IGF1] and ‐2 [IGF2], C‐type natriuretic peptide [CNP], and bone metamorphic protein 7 [BMP7] have been shown to have these characteristics both in vitro and in vivo. However, it is not known how these molecules compare each other regarding their effect on phenotype and stimulation of ECM production in primary chondrocytes. The effects of sprifermin, IGF1, IGF2, CNP, and BMP7 were evaluated on bovine articular chondrocytes, first in monolayer to determine their effective concentrations, and then in three‐dimensional (3D) culture at concentrations of 100 ng/ml for sprifermin; 300 ng/ml for IGF1, IGF2, and BMP7; and 10 nM for CNP. In 3D culture, the effects of a permanent exposure or a cyclic exposure consisting of 24 h incubation per week with the compounds were evaluated. All growth factors increased ECM production and cell proliferation to a similar extent but CNP had almost no effect on bovine chondrocytes. Sprifermin was more effective with cyclic exposure, IGF1, and IGF2 with permanent exposure, and BMP7 showed similar results with both exposures. Regarding the cell phenotype, sprifermin appeared to be the only compound favoring the chondrocyte phenotype; it decreased type I collagen expression and had no hypertrophic effect. Together, these results confirmed that sprifermin is a promising disease‐modifying OA drug. © 2019 The Authors. *Journal of Orthopaedic Research* ^®^ published by Wiley Periodicals, Inc. on behalf of Orthopaedic Research Society. J Orthop Res 38:653–662, 2020

Osteoarthritis (OA) is a degenerative joint disease affecting various tissues in the joint, with cartilage degradation being the hallmark of the disease. The associated symptoms include pain and limited functionality of the affected joint, resulting in a considerably reduced quality of life. The most recent Global Burden of Disease report (GBD2013) estimated that globally 242 million people are living with symptomatic and activity‐limiting OA of the hip and/or knee.^1^ Current treatment strategies include a recommended program of weight loss, muscle strengthening exercises, and non‐steroidal anti‐inflammatory drugs (NSAIDs) and hyaluronic acid or corticosteroid injections for pain management.^2^ For the more severe cases, a surgical intervention consisting of total joint replacement may be required. Despite the prevalence of OA, there is a lack of available disease‐modifying OA drugs (DMOADs) that can slow or stop the degradation of cartilage and reduce symptoms.

The two main target strategies for potential DMOADs are either anti‐catabolic (which aims to stop the process of cartilage degradation) or pro‐anabolic (which aims to stimulate the growth of new, healthy cartilage). Sprifermin, a recombinant human fibroblast growth factor 18 (rhFGF18), is a potential pro‐anabolic DMOAD that is currently in clinical development for knee OA.^3^ In in vitro studies with OA human chondrocytes three‐dimensional (3D) culture, sprifermin has been shown to stimulate chondrocyte proliferation, favor the chondrocyte phenotype, and stimulate accumulation of a hyaline extracellular matrix (ECM).^4^ In clinical trials, treatment with sprifermin increased cartilage thickness in patients with knee OA.^3^, ^5^

Several other growth factors have been tested for their anabolic properties on cartilage or chondrocytes. Insulin‐like growth factor 1 (IGF1) and 2 (IGF2) are proteins with a similar structure to insulin. IGF1 has been shown to stimulate cell proliferation and to increase proteoglycan and collagen production in vitro, and to promote cartilage repair in vivo.^6^, ^7^, ^8^, ^9^ The anabolic effect of IGF2 on chondrocytes or cartilage has been less extensively investigated but there are reports that IGF2 enhanced matrix levels in human OA cartilage explant culture and ameliorated OA in vivo.^10^

BMP7, a member of the bone metamorphic proteins (BMPs) is another well‐characterized cartilage anabolic factor. BMP7 has been shown to stimulate the production of proteoglycan^11^ and the expression of aggrecan and type II collagen^12^ in vitro, and to inhibit OA progression in vivo.^13^ BMP7 entered clinical development^14^, ^15^ but no efficacy data have been reported.

Finally, C‐type natriuretic peptide (CNP) is a natriuretic peptide involved in cartilage homeostasis.^16^ CNP has been found to stimulate cell proliferation at concentrations of 10–100 pM and matrix deposition at 10 nM in bovine chondrocytes.^17^ Furthermore, overexpressing CNP in chondrocytes in the K/BxN TCR mouse model of arthritis was shown to reduce cartilage damage.^18^

Alongside their chondrocyte‐anabolic properties, none of these compounds (IGF1, IGF2, CNP, sprifermin, and BMP7) appear to exert any deleterious effects on other tissues in the joint. This is in contrast to some other agents with chondrocyte‐anabolic properties, such as FGF2, transforming growth factor β (TGF‐β), and BMP2, which were therefore excluded from this study.^19^ Interestingly, only sprifermin and BMP7 have progressed into clinical trials of knee OA. One potential reason for this could be greater efficacy of sprifermin and BMP7 in stimulating hyaline cartilage growth compared with IGF1, IGF2, and CNP. However, it is difficult to directly compare these anabolics based on results from published literature because different culture systems or cells were used. Therefore, the present study aimed to directly compare the efficacy of IGF1, IGF2, CNP, sprifermin, and BMP7 in the same cell type and the same culture system. Initially, a monolayer culture was performed with increasing doses of the tested molecules in order to determine the most effective dose of each anabolic on bovine chondrocytes. Thereafter, the 3D culture was performed with a dose chosen from the monolayer experiment. The 3D culture was also used to investigate whether intermittent or permanent exposure to each anabolic produces different effects, as shown previously with sprifermin.^4^ The goal of this second part of the study was to better compare the quality and quantity of the ECM produced by chondrocytes exposed to these anabolics and compare their effect on the chondrocyte phenotype.

## METHODS

### Anabolic Compounds

Sprifermin, a recombinant human FGF18, was expressed in *Escherichia coli* and purified as previously described.^20^ Sprifermin is a truncated, 170 amino acid form of FGF18 (MW = 19.83 kDa), from which the signal sequence, and the 11 C‐terminal amino acids, have been removed. Consequently, sprifermin starts with a methionine followed by amino acid 28 (Glu) and ends with amino acid 196 (Lys) of the wild‐type human FGF18.

BMP7 was obtained from R&D System (Minneapolis, MN), IGF1 and IGF2 were from Merck‐Millipore (Darmstadt, Germany) and CNP from Sigma‐Aldrich (St. Louis, MI). All protein produced in a cell system had low levels of endotoxin (<0.1EU/µg). CNP was chemically synthesized.

### Chondrocyte Isolation and Culture

Bovine chondrocytes were isolated from the metacarpal joint of cattle aged 1–2 years, collected from a local slaughterhouse. The cartilage was digested in 0.1% (w/v) collagenase NB4 (Serva, Heidelberg, Germany) at 37°C overnight and the resulting cell suspension was subsequently filtered, washed, and resuspended in a culture medium with 1% penicillin and streptomycin. The culture medium was composed of Dulbecco's modified Eagle's medium (DMEM) High Glucose media (Thermo Fisher Scientific, Waltham, MA) supplemented with 10% fetal bovine serum (Biochrom, Berlin, Germany), 50 µg/ml ascorbate‐2‐phosphate (Sigma‐Aldrich), and 0.4 mM proline (Sigma‐Aldrich).

For the monolayer culture, the cells were first cultured for 1 week in the culture medium. The cells were then harvested and seeded at 15,000 cells per well in a 24‐well plate and cultured in culture medium with 0.3–1,000 ng/ml sprifermin, BMP7, IGF1, and IGF2, or 0.003–10 µM CNP. Two monolayer cultures were performed; one with IGF1, IGF2, and CNP and a second one with BMP7 and sprifermin. After 7 days, the medium was harvested for glycosaminoglycan (GAG) analysis and the cells were detached using accutase (Sigma‐Aldrich) containing 2.5% collagenase. The cells were counted with a Vi‐cell™ XR cell counter (Beckman Coulter, Krefeld, Germany) or processed for gene expression.

For the 3D culture, freshly isolated chondrocytes were used and seeded at 1 × 10^6^ cells per well in 96‐well ultra‐low binding plates and cultured for first 1 week in a culture medium without compound and subsequently 4 weeks with IGF1, IGF2, or BMP7 at 300 ng/ml, sprifermin at 100 ng/ml, or CNP at 10 nM. Two different experiments were performed; one with sprifermin, IGF1, and IGF2 and another one with sprifermin, CNP and BMP7. In both experiments, the 3D constructs were incubated with the compounds either permanently or intermittently (1 day per week; cyclic exposure). At the end of the culture period, the 3D constructs were either used for the analysis of the GAG and hydroxyproline (HPro) content, gene expression, or histological analysis. Prior to biochemical analysis, the constructs were digested overnight at 60°C with papain 0.125 mg/ml (Merck KGaA, Darmstadt, Germany) in 0.1 M Na_2_HPO_4_, 0.01 M ethylenediaminetetraacetic acid, and 5 mM l‐cysteine. All cultures were done with *N* = 3–4.

### Biochemical Measurements

GAG was measured with the DMMB (dimethyl methylene blue) assay as previously described.^4^ The HPro measurement was performed using a high‐performance liquid chromatography‐mass spectrometry/mass spectrometry (HPLC‐MS/MS) assay, using 4‐hydroxyproline (VWR International, Radnor, PA) 0.1–50.0 µg/ml as calibration standards. Then, 5 µl of the samples were mixed with 10 µl of internal standard (1.2 µg/ml 4‐hydroxyproline [^2^H_3_] from C/D/N‐Isotopes) and 200 µl of 25% (v/v) hydrochloric acid and hydrolyzed overnight at approximately 110°C. After centrifugation, samples were evaporated at 55°C/10 Torr, resuspended in 1 ml water and mixed 1:5 in acetonitrile. The HPLC separation (1200 Series HPLC, Agilent, Santa Clara, CA; HTC PAL Autosampler, CTC Analytics, Zwingen Switzerland) was achieved on a hydrophilic interaction chromatography column (Sequant ZIC‐HILIC, 50‐2.1 mM, 3.5 µM, 200 Å column; Merck KGaA) using a mobile phase gradient (eluent A: 0.1% [v/v] formic acid in water, eluent B: acetonitrile). Detection was performed on a tandem MS (API4000; Sciex, Darmstadt, Germany) with a turbo ion spray interface operating in positive ion mode.

### Gene Expression

RNA isolation was performed with the RNeasy Mini Kit (Qiagen, Hilden, Germany) according to the recommendation of the manufacturer. The 3D constructs were additionally treated with proteinase K (Qiagen) for 10 min at 55°C before RNA isolation. The messenger RNA (mRNA) concentration and quality were analyzed using an Agilent Bioanalyzer (Model 2100, Agilent) with an Agilent RNA 6000 Nano Chip G2938‐80023.

Reverse‐transcription was performed with the SuperScript III First‐Strand Synthesis SuperMix (Invitrogen, Carlsbad, CA) and followed by an RNAse H treatment. Quantitative polymerase chain reaction (PCR) was performed with the SYBRGreen Jumpstart Taq Ready Mix (Sigma‐Aldrich) with 200 nM of the reverse and forward primer (Eurofins Genomics, Ebersberg, Germany; see Table 1) in the thermocycler Mx3000P (Agilent).

**Table 1:** Primers Used for Quantitative Polymerase Chain Reaction (qPCR)

Genes	Forward Sequence	Reverse Sequence
EF1a	AGCTGAAGGAGAAGATTGATC	GGCAGACTTGGTGACCTTG
Col1a2	TGGCCCAGAAGAACTGGT	AGGAAGGTCAGCTGGATG
Col2a1	GAACCCAGAACCAACACAATCC	TCTGCCCAGTTCAGGTCTCTTAGAGA
ACAN	GAAACCTCTGGACTCTTTGGTGTC	GCCAGATATTTCTCCATAAAACCCTGA
MMP13	CCCCTACTCTAAACATCCCAAAAC	AACAGCTCTGCTTCAACCTG
Runx2	TCATCTGGCTCAGGTACGAG	TCATCTGGCTCAGGTACGAG
Col10a1	TTTCCCCTTTCTGTCCATTC	AGCTGGTTTACCAGGACCAC

For each gene, the cycle threshold (*C*_t_) was determined and the relative abundance was calculated according to the following formula:

$relative\_abundance=2^{\left(ctHKG-ctGOI\right)}=\frac{2^{ctHKG}}{2^{ctGOI}}$ where HKG = house‐keeping gene (*eef1a1*, eukaryotic elongation factor 1α) and GOI = gene of interest (*col1a2, col2a1, acan, mmp13, runx2*, or *col10a1*).

### Histology

The samples were embedded in paraffin and 5 µm slices were cut with a rotary microtome. The standard staining was Safranin O 0.01% (w/v) with Fast Green 0.1% (v/v) as a counterstain. The immunohistochemical detection of type I and II collagens was achieved using a fully automated immunohistochemistry stainer (Bond‐III; Leica, Wetzlar, Germany). For type I collagen staining, antigen retrieval was performed with proteinase K 0.61 µg/ml (Leica) for 5 min at 37°C and the samples stained with a rabbit anti‐collagen I antibody at 0.7 µg/ml (ab6308; Abcam, Cambridge, UK) followed by the Bond Polymer Refine Detection kit (Leica). For type II collagen staining, antigen retrieval was performed with chondroitinase ABC 0.5 U/ml (Sigma‐Aldrich) for 15 min at 37°C and the samples stained with a rabbit anti‐collagen II antibody (ab34712; Abcam) at 2.5 µg/ml followed by the EnVision + System‐HRP detection kit (Agilent).

### Statistical Analysis

Statistical analysis was conducted using Graphpad Prism v7.00 (GraphPad Software, San Diego, CA). One‐way analysis of variance was performed with a Dunnet test to correct for multiple comparisons.

## RESULTS

### Effects of IGF1, IGF2, CNP, BMP7, and Sprifermin in Monolayer

**Figure 1:**
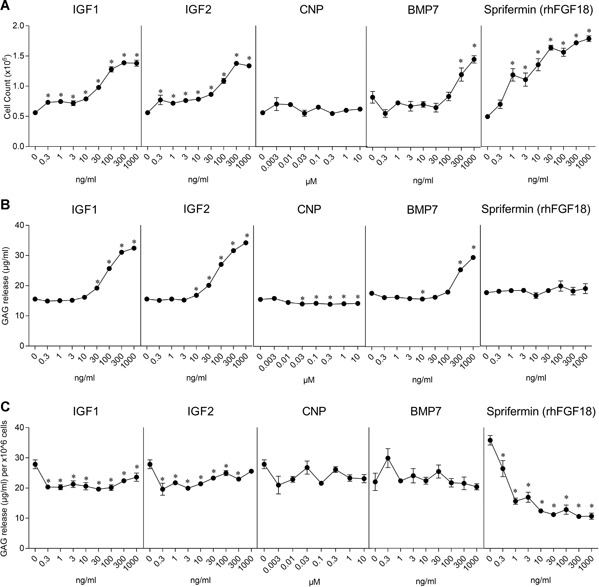
Dose‐dependent effect of insulin‐like growth factor‐1 (IGF1), IGF2, C‐type natriuretic peptide (CNP), bone metamorphic protein 7 (BMP7), and sprifermin on chondrocytes in monolayer. Two monolayer cultures were performed; one with IGF1, IGF2, and CNP and a second one with BMP7 and sprifermin. (A) Cell number, (B) glycosaminoglycan (GAG) content of media, and (C) relative GAG production after 7 days of stimulation with increasing concentrations of the molecules. Data represent the mean ± standard error of the mean (SEM). *p < 0.05 versus control (culture medium only).

The cells were treated with 0.3–1,000 ng/ml of IGF1, IGF2, BMP7, or sprifermin and 0.003–10 µM of CNP for 7 days; and the cell number, GAG production, and gene expression were measured. The cell number (Fig. 1A) increased in a dose‐dependent manner with the four growth factors but was unchanged across all concentrations in the CNP‐treated samples. At the highest concentration, the cell number was 2.5‐fold higher than in the control with IGF1, 2.4‐fold higher with IGF2, 1.8‐fold higher with BMP7, and 3.6‐fold higher with sprifermin. IGF1, IGF2, and BMP7 also stimulated an increase in GAG release in a dose‐dependent manner (Fig. 1B); at the highest concentration, GAG release was 2.1‐fold higher than in the control with IGF1, 2.2‐fold higher with IGF2, and 1.7‐fold higher with BMP7. Sprifermin had no impact on GAG release at any dose while GAG release was slightly decreased with CNP at doses 0.03–10 µM. By normalizing GAG release to cell number, the production of GAG per million cells could be estimated. This shows (Fig. 1C) that for IGF1, IGF2, and BMP7 the increase in GAG release was due to the increased number of cells and not an increase in the GAG production in the cells. Sprifermin showed a steady dose‐related decline in the relative level of GAG release, in line with previous findings.^4^

**Figure 2:**
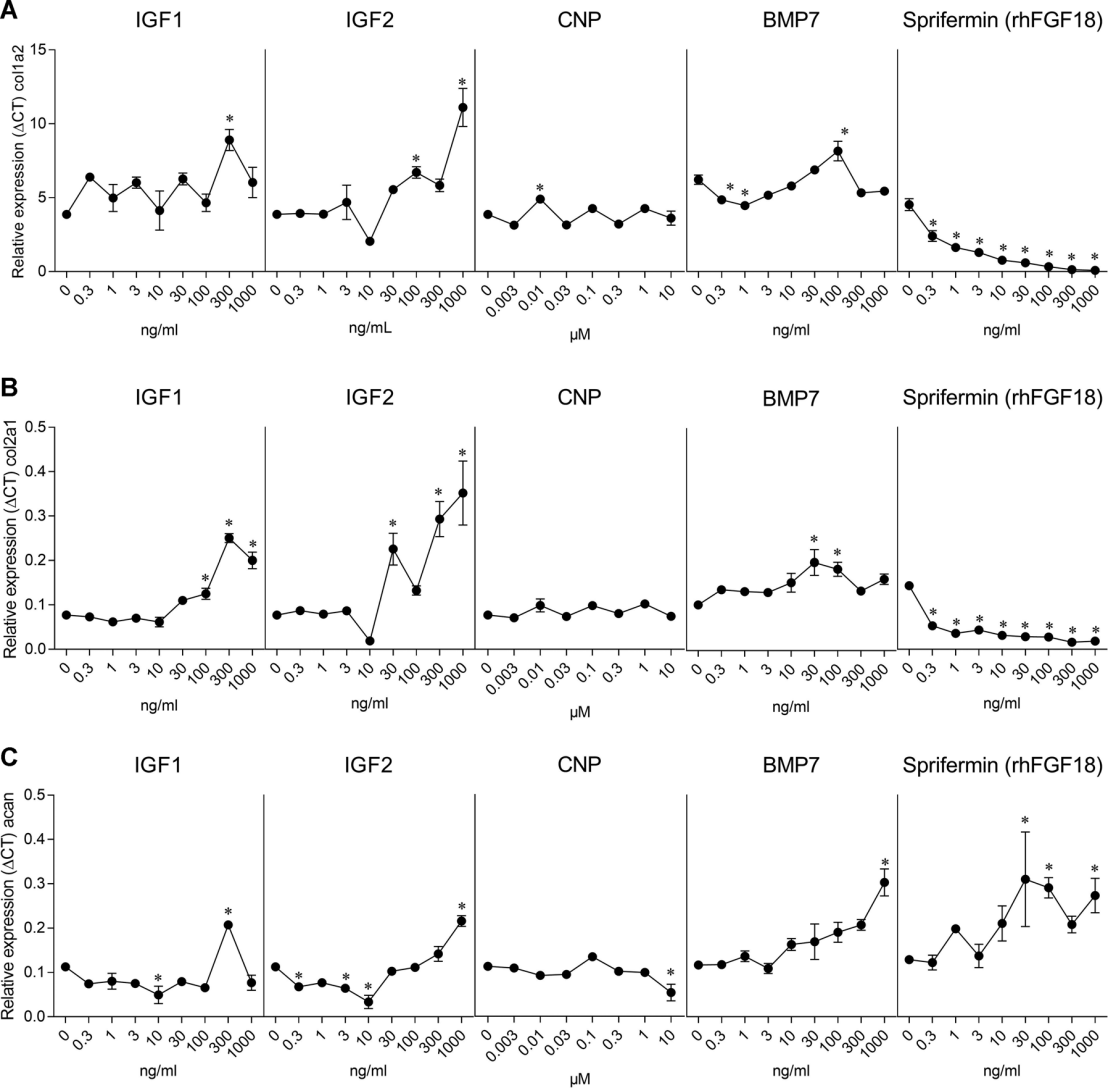
Dose‐dependent effect of insulin‐like growth factor‐1 (IGF1), IGF2, C‐type natriuretic peptide (CNP), bone metamorphic protein 7 (BMP7), and sprifermin on chondrocytes in monolayer. Two monolayer cultures were performed; one with IGF1, IGF2, and CNP and a second one with BMP7 and sprifermin. Relative expression of (A) *col1a2*, (B) *col2a1*, and (C) *acan* after 7 days of stimulation with increasing concentrations of growth factors. Data represent the mean ± standard error of the mean (SEM). *p < 0.05 versus control (culture medium only).

The expression levels of the cartilage‐related genes *col1a2, col2a1*, and *acan* in bovine chondrocytes in monolayer were evaluated (Fig. 2). Sprifermin showed a decrease in the expression of both *col1a2* (by 59‐fold at 1,000 ng/ml) and *col2a1* (by 7.6‐fold at 1,000 ng/ml), and an increase in *acan* expression (by 2.1‐fold at 1,000 ng/ml). Although the effects of IGF1 on gene expression were variable across doses, *col1a2* and *col2a1* and *acan* expression increased compared with the control (by 2.3‐, 3.2‐, and 1.8‐fold, respectively, at 300 ng/ml). Similarly, treatment with IGF2 also increased the expression of *col1a2* and *col2a1* and *acan* (by 2.9‐, 4.5‐, and 2‐fold, respectively, at 1,000 ng/ml). BMP7 stimulated a significant increase in *col1a2* (by 1.3‐fold at 100 ng/ml) and *col2a1* (by twofold at 30 ng/ml) expression, but not at concentrations above 100 ng/ml. In contrast, the highest concentration of BMP7 (1,000 ng/ml) stimulated a 2.6‐fold increase of *acan* expression. As with the cell number and GAG production, CNP treatment had little to no impact on the expression of cartilage‐related genes.

### Anabolic Effect of IGF1, IGF2, CNP, BMP7, and sprifermin in Scaffold‐Free 3D Constructs

**Figure 3:**
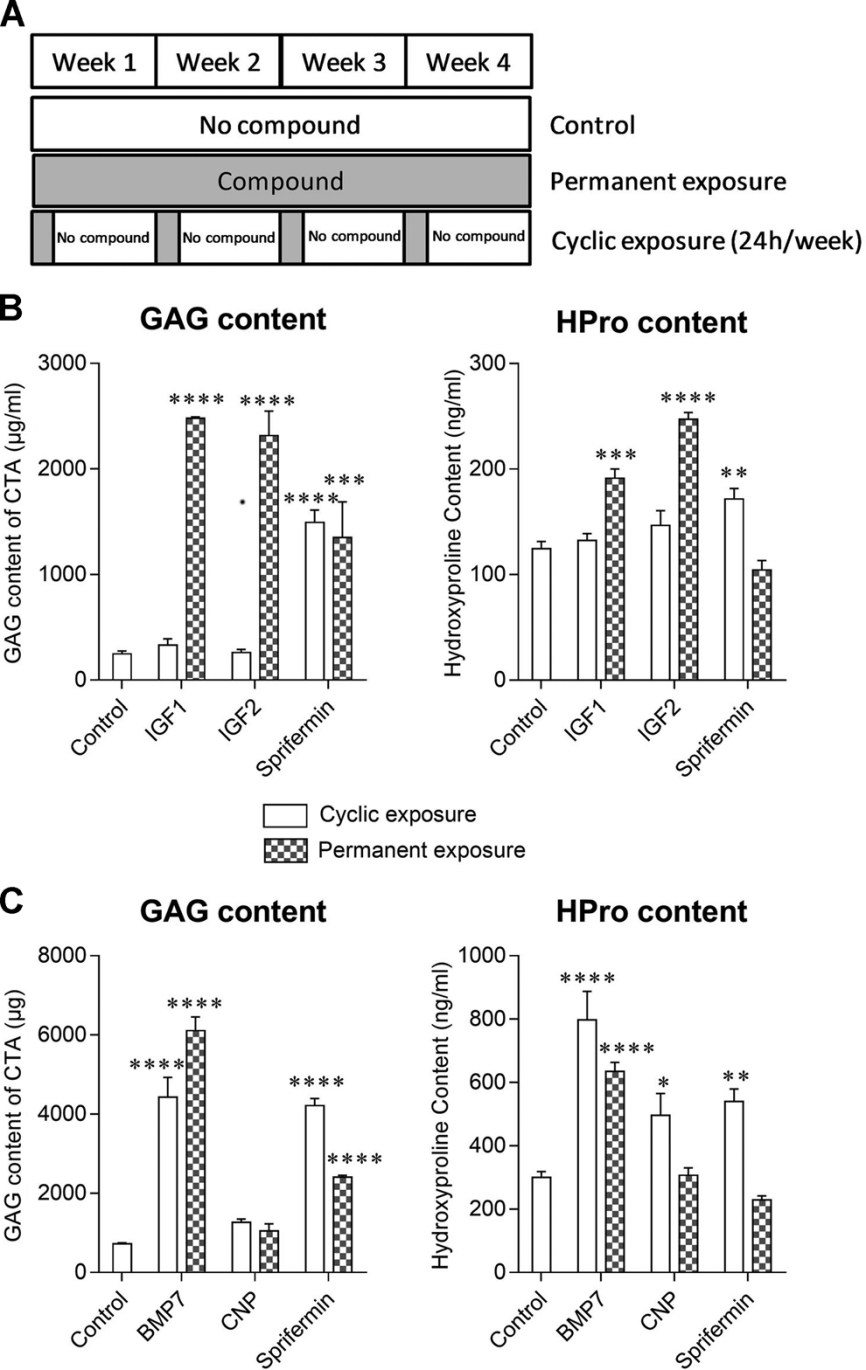
Biochemical analysis of chondrocytes in three‐dimensional (3D) constructs. (A) The 3D constructs were cultured with cyclic or permanent exposure. (B) After 4 weeks in culture with insulin‐like growth factor‐1 (IGF1), IGF2, or sprifermin or (C) with bone metamorphic protein 7 (BMP7), C‐type natriuretic peptide (CNP), or sprifermin the glycosaminoglycan (GAG) and HPro content were analyzed. Data represent the mean ± standard error of the mean (SEM). *p ≤ 0.05; **p ≤ 0.01; ***p ≤ 0.001; ****p ≤ 0.0001 versus control (culture medium only).

To further compare the anabolic functions of IGF1, IGF2, BMP7, and CNP with that of sprifermin, bovine chondrocytes were cultured in a scaffold‐free 3D construct. For IGF1, IGF2, and BMP7, 300 ng/ml was used and for sprifermin, 100 ng/ml. This corresponded to the concentrations that elicited a substantial effect on cell proliferation and GAG release in monolayer. For CNP, 10 nM was chosen based on the previous results from others.^17^ These constructs were cultured for 4 weeks with either cyclic (1 day/week) or permanent exposure to the compounds (Fig. 3A).

In both experiments (Fig. 3B and C), sprifermin significantly increased the GAG content compared with the control, with both the cyclic (by 5.5‐ or 5.8‐fold) or permanent (by 6‐ or 3.3‐fold) exposure, while the HPro content was only significantly increased with the cyclic exposure (by 1.4‐ or 1.8‐fold). The cyclic exposure to both IGF1 and IGF2 had no effect on matrix production but permanent exposure significantly increased the content of GAG (by 10‐ and 9.3‐fold, respectively), and HPro (by 1.5‐ and 2‐fold, respectively). With both the cyclic and permanent exposure, BMP7 significantly increased GAG (by 6.1‐ and 9.1‐fold, respectively) and HPro (by 2.7‐ and 2.1‐fold, respectively). CNP did not affect the GAG content but significantly increased the HPro content (by 1.7‐fold) with cyclic exposure.

**Figure 4:**
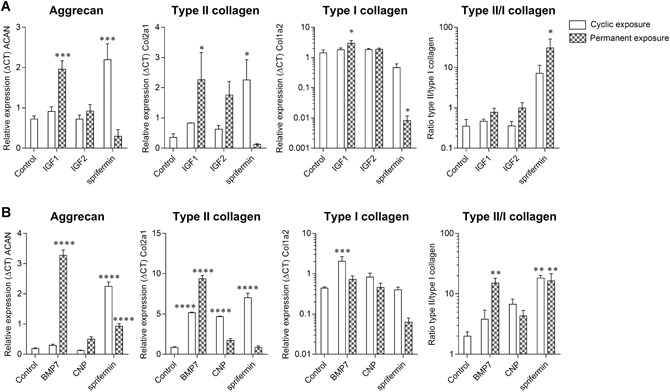
Gene expression of chondrocytes in three‐dimensional (3D) constructs. Relative expression of *acan, col2a1, col1a2*, and ratio *col2a: col1a2* from 3D constructs after 4 weeks of culture with cyclic or permanent exposure to (A) Insulin‐like growth factor 1 (IGF1), IGF2, or sprifermin or (B) bone metamorphic protein 7 (BMP7), C‐type naturetic peptide (CNP), and sprifermin. *p ≤ 0.05; **p ≤ 0.01; ***p ≤ 0.001; ****p ≤ 0.0001 versus control (culture medium only).

Analysis of cartilage gene expression from the 3D constructs is shown in Figure 4. In both experiments (Fig. 4A and B), cyclic exposure to sprifermin significantly increased the expression of *acan* (by 3‐ and 12‐fold) and *col2a1* (by 7.1‐ and 8.3‐fold) compared with the control. *Acan* expression was also observed to increase significantly with permanent exposure to sprifermin (by fivefold) in the second experiment while *col2a1* expression was unaffected by the permanent exposure to sprifermin. Permanent exposure to sprifermin decreased *col1a2* expression compared with the control (by 178‐fold in the first experiment and sevenfold in the second experiment). As a result, sprifermin increased the ratio of *col2a1:col1a2* compared with the control with both cyclic (by 20.6‐ or 18.4‐fold in the first and second experiments, respectively) and permanent exposure (by 8.8‐ or 16.5‐fold). Cyclic exposure to IGF1 and IGF2 had no effect on the expression of *acan, col2a1*, or *col1a2*. However, with permanent exposure IGF1 significantly increased *acan* (by 2.7‐fold), *col2a1* (by 7.3‐fold), and *col1a2* (by 7.4‐fold) expression. IGF2 showed a similar profile although the differences compared with the control were not statistically significant. Cyclic exposure to BMP7 significantly increased the expression of *col2a1* (6.1‐fold) and *col1a2* (by 4.6‐fold) while permanent exposure significantly increased *acan* (by 17.6‐fold) and *col2a1* (by 11.1‐fold) expression compared with the control. Permanent exposure to BMP7 also showed an increase in the ratio of *col2a1:col1a2* (by 7.5‐fold), similar to that observed with sprifermin. CNP had very few effects on gene expression; only *col2a1* expression was increased (by 5.6‐fold with cyclic exposure).

### Hypertrophic Marker Expression With IGF1, IGF2, CNP, BMP7, and Sprifermin in Scaffold‐Free 3D Constructs

**Figure 5:**
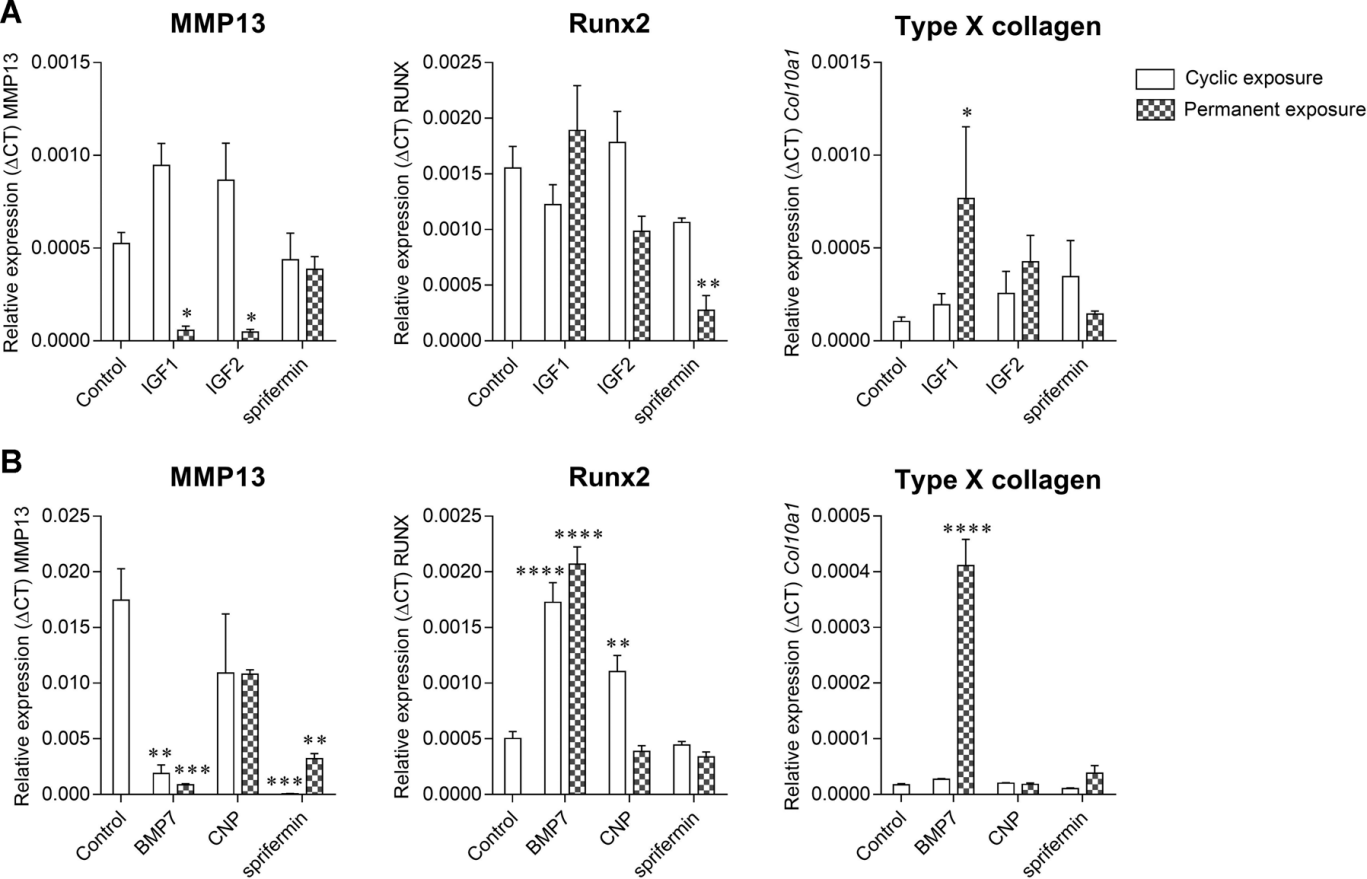
Gene expression of chondrocytes in three‐dimensional (3D) constructs. Relative expression of *mmp13, runx2*, and *col10a1* in 3D constructs after 4 weeks of culture with cyclic or permanent exposure to (A) Insulin‐like growth factor 1 (IGF1), IGF2, or sprifermin or (B) bone metamorphic protein 7 (BMP7), C‐type natriuretic peptide (CNP), and sprifermin. *p ≤ 0.05; **p ≤ 0.01; ***p ≤ 0.001; ****p ≤ 0.0001 versus control (culture medium only).

The expression of three hypertrophy markers, *mmp13, runx2*, and *col10a1*, was evaluated (Fig. 5). Sprifermin did not stimulate the expression of any of the hypertrophy markers and rather tend to decrease their expression compared with the control. *Runx2* was significantly reduced (by 5.7‐fold) after permanent exposure to sprifermin in the first experiment while *mmp13* expression was significantly reduced with both permanent (by 5.4‐fold) and cyclic (by 249‐fold) exposure in the second experiment. IGF1 and 2 both decreased significantly *mmp13* expression with permanent exposure (by 9.2‐ and 11‐fold, respectively) and had no impact on *runx2* expression. IGF1 but not IGF2 significantly increased *col10a1* expression (by 7.4‐fold) with permanent exposure. In contrast to IGFs and sprifermin, BMP7 significantly increased *runx2* expression with both cyclic and permanent exposure (by 3.4‐ and 4.1‐fold, respectively). BMP7 also significantly increased *col10a1* expression (by 24‐fold) with permanent exposure and significantly reduced *mmp13* expression with both the cyclic and permanent exposures (by 9.3‐ and 20.2‐fold, respectively). CNP did not alter *mmp13* or *col10a1* expression but cyclic exposure significantly increased *runx2* (by 2.2‐fold).

### Histological Staining of 3D Constructs

**Figure 6:**
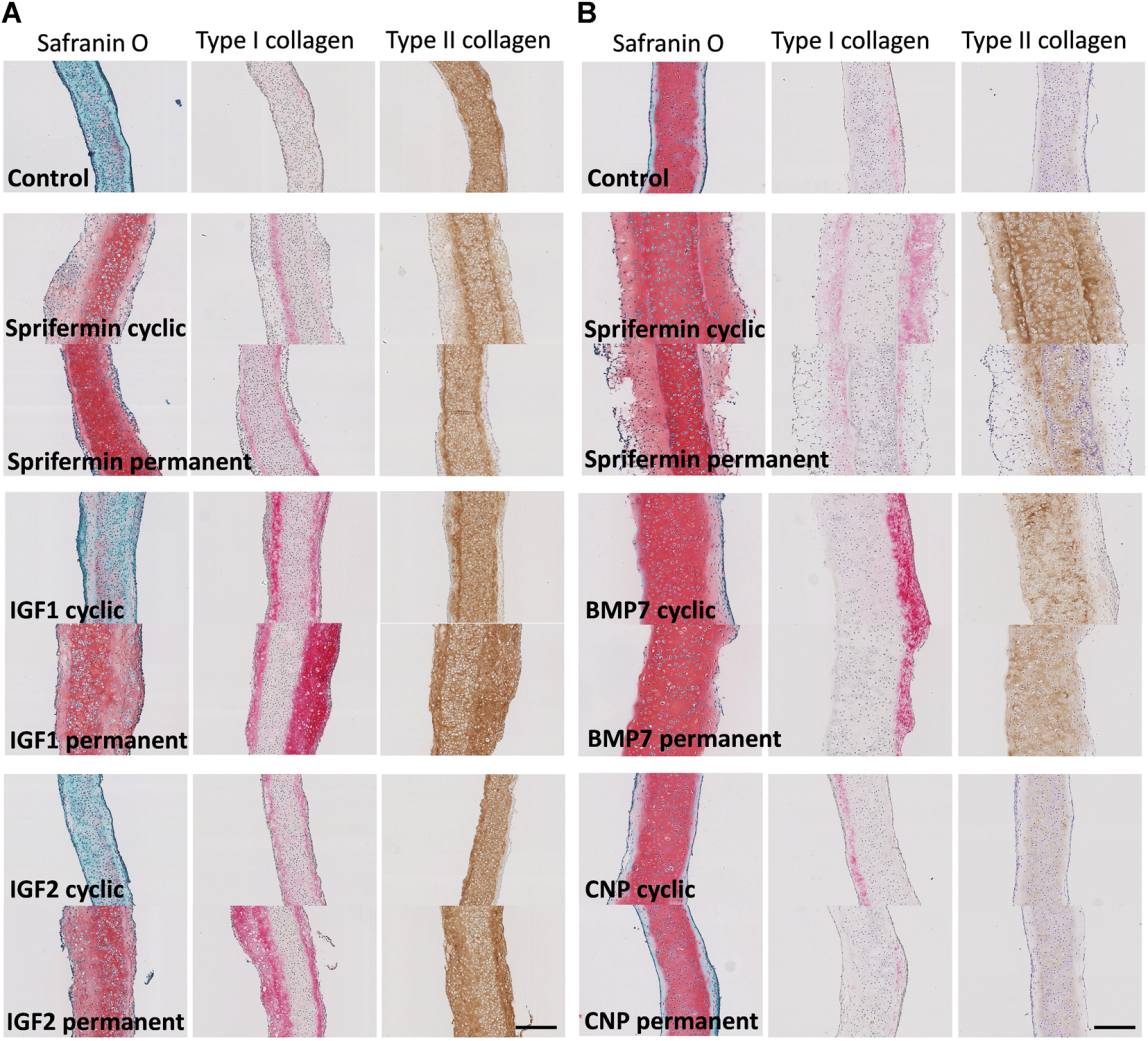
Histology of the three‐dimensional (3D) constructs after 4 weeks of culture with cyclic or permanent exposure to (A) insulin‐like growth factor‐1 (IGF1), IGF2, or sprifermin or (B) bone metamorphic protein 7 (BMP7), C‐type natriuretic peptide (CNP), and sprifermin. Safranin O/Fast green, type I and type II collagen stainings were used. Scale bar: 200 µm. [Color figure can be viewed at wileyonlinelibrary.com]

Safranin O staining and staining for type I and II collagen of the 3D constructs was performed (Fig. 6). The untreated constructs from the two cultures had different matrix compositions. In the first experiment, the constructs stained weakly for Safranin O but were strongly positive for type II collagen (Fig. 6A) but it was the opposite for constructs from the second experiment (Fig. 6B). In both cases only a faint type I collagen staining could be observed. In both cultures, cyclic or permanent exposure to sprifermin displayed a strong Safranin O and type II collagen staining while type I collagen staining stayed weak. In addition, cyclic exposure appeared to be more effective than permanent exposure (the constructs were either bigger or more homogeneously stained). The constructs treated with a cyclic exposure to IGF1 and IGF2 showed no difference in comparison with the control, but permanent exposure clearly resulted in a stronger Safranin O and type I collagen staining. Constructs that were permanently exposed to IGF1 or IGF2 also appeared to be bigger than the control. The constructs that were exposed to BMP7 (both cyclic and permanent) were much larger than the control but showed a similarly strong Safranin O staining and were similarly positive for type II collagen. However, the type I collagen staining in the BMP7‐exposed constructs appeared to be stronger than in the control. Constructs treated with CNP were not different to the control. Type X collagen staining was also performed and was negative in all conditions (not shown) despite the increased *col10a1* expression observed with permanent exposure to BMP7 or IGF1. It is possible that the gene expression was not high enough to translate into detectable type X collagen deposition.

## DISCUSSION

The treatment of osteoarthritic joints with an anabolic compound could drive regeneration and regrowth of the cartilage tissue, which is usually degraded as the disease progresses. The aim of this study was to compare the anabolic properties of several compounds reported to have positive effects on cartilage tissue—IGF1, IGF2, BMP7, and CNP—with those of sprifermin, a human recombinant FGF18, which is in clinical development for knee OA.

The anabolic compounds were first tested on bovine chondrocytes in monolayer and all of them except CNP increased cell proliferation. Furthermore, IGF1, IGF2, and BMP7 stimulated GAG accumulation in the culture medium as well as *col2a1* and *acan* expression. This is in accordance with the previously reported results.^10^, ^12^, ^21^, ^22^ *Col1a2* expression was also enhanced by these three growth factors. Sprifermin had a different profile; it induced a strong reduction of *col1a2* expression by 59‐fold at the highest dose and a more moderate reduction of *col2a1* expression by 7.6‐fold. GAG production was significantly reduced. Sprifermin was also the molecule that promoted cell proliferation the most, indicating as proposed before, that a strong proliferation might occur at the expense of matrix production.^4^ Finally, CNP had only a weak impact on chondrocytes.

For the subsequent evaluation in the 3D culture, IGF1, IGF2, and BMP7 were used at 300 ng/ml, a concentration that elicited a substantial effect on proliferation and GAG accumulation in monolayer. Sprifermin was used at 100 ng/ml, a concentration that led to a strong proliferation and demonstrated a significant anabolic effect in 3D culture in previous studies.^4^ Despite its lack of effect in monolayer, it was decided to evaluate in CNP in the 3D culture as well at a concentration of 10 nM, based on findings from others.^17^

In 3D culture, both permanent and cyclic exposure to sprifermin induced an anabolic response but the response was stronger with cyclic exposure. In addition, the ratio of *col2a1:col1a2* was increased with both exposures, while the expression of hypertrophy markers was unaffected, indicating that sprifermin favored the chondrocyte phenotype. These results confirmed similar findings obtained with porcine and human OA chondrocytes in the same culture model.^4^

IGF1 and IGF2 both displayed a similar profile in 3D culture; cyclic exposure had no impact, but the permanent exposure resulted in an increased ECM production. The anabolic effect observed with permanent exposure is in accordance with the previous studies.^6^, ^7^, ^10^ However, although permanent exposure to IGF1 elevated *col1a2* as previously reported,^23^ none of the IGFs had an effect on the *col2a1:col1a2* ratio. Regarding the hypertrophy markers, both IGFs decreased *mmp13* expression as already described,^10^, ^24^ but IGF1 was found to increase *col10a1* expression with permanent exposure.

Similar to other reports,^11^, ^23^, ^25^ BMP7 had anabolic effects with permanent exposure, significantly increasing the expression of *acan* and *col2a1* but having no effect on *col1a2*. Interestingly, the cyclic exposure to BMP7 did elicit an increase of the expression of *col2a1* but not *acan*, and in this case, *col1a2* was elevated. It was also previously reported that BMP7 has no hypertrophic effect in bovine articular chondrocytes and may even have a hypertrophy‐suppressive effect in ATDC5 and human bone marrow stem cells of BMP7.^26^, ^27^ On the contrary, our results show that BMP7 tended to increase expression of some hypertrophy markers.

Finally, CNP had almost no effect in 3D culture similar to that observed in the monolayer culture. In contrast to our findings, Waldman et al.,^17^ demonstrated that CNP stimulates proliferation and matrix production in bovine chondrocyte in a 3D culture at the same concentration as used in the present study (10 nM). However, we used a CNP from another supplier and it cannot be excluded that the bioactivity might differ from one supplier to the other.

This head‐to‐head comparison of sprifermin, IGF1, IGF2, and BMP7 enables direct comparison of the effects of these growth factors on the phenotype and anabolism of primary articular chondrocytes to be made. To our knowledge, it is also the first study where IGF1, IGF2, and BMP7 have been evaluated with a cyclic exposure compared with a permanent exposure. When comparing the four growth factors in terms of the chondrocyte phenotype, it appeared that sprifermin was the only one that decreased *col1a2* expression both in monolayer and in 3D with permanent exposure. On the contrary, both IGFs and BMP7 increased *col1a2* expression in monolayer and in 3D and displayed a stronger type I collagen staining compared with sprifermin. Furthermore, sprifermin did not increase the expression of any of the hypertrophy markers.

In terms of their anabolic effect, the four growth factors were equivalently effective: sprifermin with cyclic exposure, IGFs with permanent exposure and BMP7 with both exposures. However, showing efficacy with a cyclic exposure is of great advantage for potential OA therapy.

OA is a localized disease and many therapeutic approaches use the local application, namely intra‐articular injection.^28^ Intra‐articular injections have the advantages that the compound is injected directly where it is needed. In addition, low systemic exposure decreases the probability of unwanted side‐effects. However, it has disadvantages as well: retention time in the joint capsule is limited and injection can only be performed with reasonable intervals. Molecules displaying an anabolic effect with a cyclic exposure increases their applicability for OA, as occasional injections might be enough to trigger a therapeutic response. Indeed, in a randomized, double‐blind, placebo‐controlled Phase II study, sprifermin was administrated intra‐articularly to patients with knee OA once weekly for 3 weeks followed by a 5‐month break before starting a new treatment cycle. This dosing regimen leads to an increased cartilage thickness at Year 2.^5^ BMP7, which might also be compatible with cyclic exposure, has been tested in the clinic. Initial results from a Phase II study described potential anabolic effects but the final results, as well as further development, was not communicated.^15^ Finally, IGF1 and IGF2 did not reach clinical development for OA. In a rat medial meniscal tear model, it was demonstrated that intra‐articular IGF1 had no effect on cartilage degeneration but that a modified heparin‐binding IGF1 with increased retention in cartilage ameliorated OA.^8^ These findings are in accordance with the need for prolonged exposure to IGF1.

This study presents some limitations. For instance, it is unclear how results obtained with bovine chondrocytes translate to the human situation; however, testing many conditions with human chondrocytes would have been challenging due to the low availability of this material. Another limitation is that only one independent experiment was realized for each culture condition and compound. However, we believe that testing the same molecules in different settings (monolayer with permanent exposure, 3D culture with permanent and cyclic exposure) enabled us to robustly compare the tested molecules.

In conclusion, sprifermin and BMP7, but not IGF1 and IGF2, exerted an anabolic effect with cyclic exposure on bovine chondrocytes. This characteristic makes them suitable for intra‐articular treatment for OA without the need for a slow‐release formulation. In addition, sprifermin was the only tested compound that favored the chondrocyte phenotype by decreasing type I collagen and having no hypertrophic effect. Consequently, sprifermin is more likely than BMP7, IGF1, or IGF2 to result in the production of hyaline cartilage. The results of this study confirm that sprifermin presents a promising therapeutic option as an intra‐articular DMOAD.

## AUTHORS’ CONTRIBUTION

S.M. designed the experiments, acquired and analyzed the data, and drafted the paper. A.G. designed the experiments, analyzed the data, and critically reviewed the paper. S.L. participated in data acquisition and critically reviewed the paper. All authors have read and approved the final submitted manuscript.
